# Complete chloroplast genome of *Gypsophila oldhamiana* Miq. (Caryophyllales: Caryophyllaceae)

**DOI:** 10.1080/23802359.2021.1997126

**Published:** 2021-11-10

**Authors:** Ji Ran Jeong, Hyun Jo Koo, Heesu Yun, Hyang Sook Chun, Sang-Il Han, Gyoungju Nah

**Affiliations:** a Genome Analysis Center at National Instrumentation Center for Environmental Management, Seoul National University, Seoul, Republic of Korea; b Department of Food Science and Technology, Chung-Ang University, Ahnsung, Republic of Korea; c Medicinal Herb Garden, College of Pharmacy, Seoul National University, Goyang, Republic of Korea

**Keywords:** *Gypsophila oldhamiana*, caryophyllaceae, complete chloroplast genome, next generation sequencing

## Abstract

The complete chloroplast genome sequence of *Gypsophila oldhamiana* Miq., a species of the *Caryophyllaceae* family, was assembled and analyzed from the *de novo* assembly of Illumina paired-end sequencing data. The total length of the chloroplast genome of *G. oldhamiana* was 152,675 bp including a large single-copy (LSC) region of 83,552 bp, a small single-copy (SSC) region of 17,349 bp, and a pair of identical inverted repeat regions (IRs) of 25,887 bp. The genome possessed a total of 130 genes, including 85 protein-coding genes, 37 transfer RNA (tRNA) genes, and 8 ribosomal RNA (rRNA) genes. The phylogenetic analysis of *G. oldhamiana* with 14 related species discovered the closest taxonomical relationship with *Gypsophila vaccaria voucher* in the Caryophyllaceae family.

*Gypsophila* belongs to the Caryophyllaceae (pink family or carnation family) and comprises about 50 species in NCBI Taxonomy, but the chloroplast genome sequence was reported from only one species, *G. vaccaria* (Yao et al. 2019). Some *Gypsophila* species have been traditionally used to treat coughs, colds, and ailments of the upper respiratory tract (Elbandy et al. 2007), and triterpene saponins from the underground parts are considered as main active compounds (Servi et al. 2019). *Gypsophila* species grow in gypsum environments, and they mainly habitat in the Mediterranean and Irano-Turanian regions (Madhani et al. 2018). *G. oldhamiana* Miq. is a perennial herbaceous plant which grows in Northeast Asia including the northern regions of China and Korea. *G. oldhamiana* has also been used as a traditional medicine to treat fever, consumptive disease and infantile malnutrition (Xie et al. 2016).

The leaves of *G. oldhamiana* were provided from Medicinal Plant Garden, College of Pharmacy, Seoul National University, Koyang, Korea (37°42'44.9"N 126°49'08.0"E). The specimen was deposited in the National Institute of Biological Resources (NIBR, https://www.nibr.go.kr/cmn/main/enMain.do, Chang woo Hyun, john0920@korea.kr) under the voucher number of NIBRVP0000823699 and a total genomic DNA extracted from leaf tissues was also deposited in NIBR (Yoon-Jeong Park, byj6019@korea.kr) with collection number of NIBRGR0000634617. Genomic DNA extracted from leaves was used to construct the genomic library for Illumina 150 bpX2 paired-end (PE) sequencing. We used the high-quality PE reads for assembly using CLC Genomics Workbench (ver. 10.0.1, CLC QIAGEN), and manually curated through mapping Illumina raw reads to the assembled contigs (Kim, Lee, et al. 2015). Using GeSeq and manual corrections, annotation of the complete chloroplast genome was performed (Tillich et al. 2017). The complete chloroplast genome sequence of *G. oldhamiana* was submitted to GenBank with the accession number MZ557565.

The complete chloroplast genome of *G. oldhamiana* was 152,675 bp in length with 36.43% of G + C content, displaying a large single copy (LSC) region of 83,552 bp, a small single copy (SSC) region of 17,349 bp, and a pair of inverted repeat (IRa and IRb) regions of 25,887 bp. The genome possessed 130 genes, including 85 protein-coding genes, 37 tRNA genes, and 8 rRNA genes. Interestingly, the product of *infA* is partial in *G. oldhamiana* chloroplast genome, and this pseudogene may indicate the gene transfer to the nucleus as occurred in diverse plant species (Millen et al. 2001).

To examine the phylogenetic position of *G. oldhamiana,* the complete chloroplast genome sequences of *G. oldhamiana* and 14 related species were aligned using MAFFT (ver. 7.271) (Katoh et al. 2002), followed by phylogenetic tree construction based on a Maximum Likelihood (ML) analysis with 1,000 bootstraps through MEGA 10.2.5 (Kumar et al. 2016). The phylogenetic analysis exhibited the close relationship of *G. oldhamiana* with *Gypsophila vaccaria* in the family of Caryophyllaceae (Figure 1).

**Figure 1.**
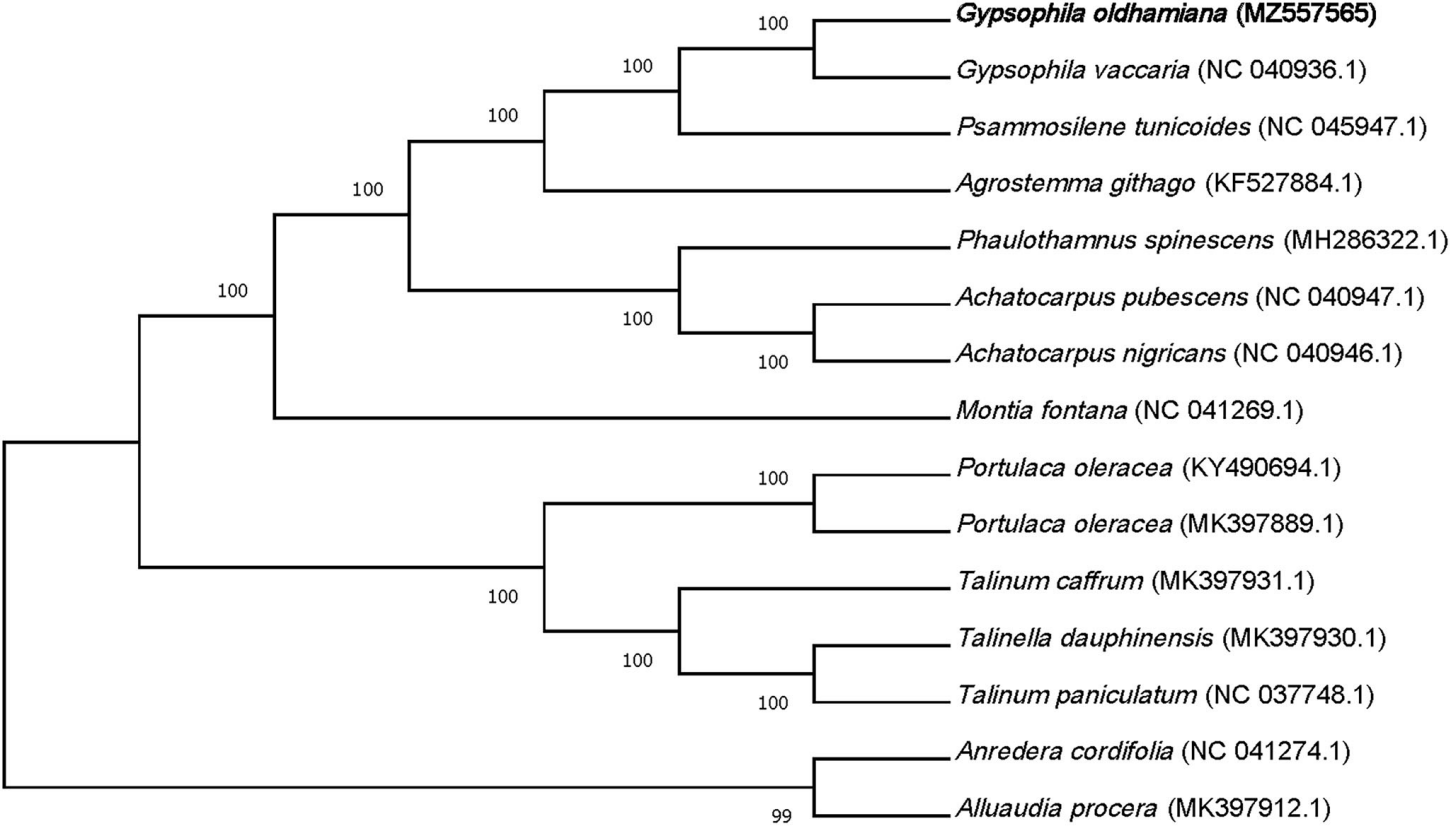
The construction of phylogenetic tree was performed using complete genomic sequences of 14 species and *G. oldhamiana* based on maximum likelihood method with a bootstrap value of 1000 replicates.

## Data Availability

The data that support the finding of this study are publically available in GenBank at http://www.ncbi.nlm.gov/genbank/, with reference number, MZ557565. The BioProject, BioSample, and SRA numbers are PRJNA750158, SAMN20445193, and SRR15275533, respectively.
